# Stay-at-home orders during COVID-19 pandemic: an experience from general population in Damietta Governorate, Egypt

**DOI:** 10.1186/s42506-022-00115-3

**Published:** 2022-10-03

**Authors:** Mohamed O. Nour

**Affiliations:** 1 Department of Public Health and Community Medicine, Damietta Faculty of Medicine, Al-Azhar University, Damietta, Egypt; 2 Department of Health Education and Promotion, Faculty of Public Health and Health Informatics, Umm Al-Qura University, Makkah, Kingdom of Saudi Arabia

**Keywords:** Stay-at-home, Social distancing, COVID-19 pandemic, Damietta, Egypt

## Abstract

**Background:**

The COVID-19 pandemic in Egypt triggered national preparedness, public engagement, and an integrated response that included social distancing measures, for example, staying at home. We aimed to investigate community awareness of and commitment to complying with the stay-at-home orders in Damietta Governorate, Egypt, during the COVID-19 pandemic.

**Methods:**

A web-based cross-sectional survey was designed and completed by 500 adult participants from Damietta, Egypt, between April 10 and July 15, 2020. Participants were asked about their sociodemographics, sources of knowledge about COVID-19, awareness of COVID-19 prevention methods, commitment to stay-at-home orders, and their trust in governmental measures, community resources, and emergency services. The participants were classified as stay-at-home responders or nonresponders.

**Results:**

Of the participants, 18.4% responded to stay-at-home orders; the main reasons for leaving home were buying essentials, especially food, and going to work. Compliance was significant among elderly individuals and those with a history of chronic illness. Nonresponse was significant among individuals who were married, working, or had low family income. More than one-third (39.2%) had good knowledge of effective methods of COVID-19 prevention, and the overall accepted knowledge was significantly higher among stay-at-home responders than nonresponders. Their trust in governmental measures, community resources, and emergency services to manage the pandemic was poor—84.6%, 71.8%, and 79%, respectively—with no significant differences between the groups.

**Conclusions:**

Participants’ compliance with and engagement in stay-at-home orders in Damietta Governorate, Egypt, was poor. Public response to stay-at-home orders is affected by sociodemographics, and the public’s trust in governmental measures, community resources, and emergency services was poor. Understanding how social distancing is perceived in Egypt is important to provide public support and improve pandemic disease containment.

## Introduction

Influenza viruses have led to high morbidity and mortality in humans and are a major public health threat. Novel influenza viruses with pandemic potential, such as avian influenza, swine flu, severe acute respiratory syndrome (SARS), and newly recognized novel coronavirus (COVID-19), are a few examples of such diseases [1].

The COVID-19 pandemic has created an unprecedented public health concern and was declared a public health emergency of international concern [2]. The World Health Organization (WHO) situation report, issued on September 5, 2021, confirmed that there had been over 218.9 million cases of COVID-19, with approximately 4.5 million deaths, with the greatest prevalence reported in the Americas. In Egypt, according to the Ministry of Health (MOH), the first confirmed case was announced on February 14, 2020; the first case in Damietta Governorate (northeast of Egypt) was announced on March 7, 2020; and the first death occurred on March 23, 2020 [3]. By September 5, 2021, in Egypt, 290,027 cases of COVID-19 were officially confirmed, resulting in 16,789 deaths [4].

Quarantine and isolation have been used as public health measures to curb COVID-19 transmission by reducing public contact rates, potentially incubating the virus, and significantly flattening the epidemic curve. However, their effectiveness is controversial, and their impact is wide ranging and can be long lasting. Depriving individuals of their liberty and autonomy for the public good is often contentious and requires careful implementation because these policies may inconvenience individuals; lead to depression, post-traumatic stress disorder, stigma, social isolation, and economic loss; or contribute to moral conflicts. In addition, quarantine and isolation can be difficult to implement because of, for example, public doubts [5, 6].

Various control measures have been announced by the Centers of Disease Control and Prevention (CDC) and the WHO to curtail the spread of the COVID-19 pandemic. Vaccination is crucial in disease control. However, before effective vaccines for COVID-19 were available, prevention was limited to non-pharmaceutical interventions, including top-down (i.e., governmental) and bottom-up (i.e., self-initiated) measures recommended by national and international agencies and government bodies, for example, bans on travel and social gatherings, curfews, lockdowns, school closures, remote working, social distancing, stay-at-home orders, and hygienic and personal protective measures [7, 8].

Early detection of COVID-19 cases requires an effective disease surveillance system that may incur operational challenges in Egypt, including the lack of a precise, timely information exchange at different levels, underreporting, and poor community support. These challenges highlight the need for community involvement in surveillance, behavioral change interventions, and efforts to accept the idea of social distancing, including staying at home, to control the spread of the disease and prevent human-to-human transmission [9].

With the unpredictable nature of the COVID-19 pandemic, virus mutations, and subsequent epidemic waves, understanding the public reaction to the widespread social distancing measures, including staying at home, is essential, and authorities should implement all appropriate measures to ensure that this experience is as tolerable as possible [10].

Therefore, factors involved in social distancing and staying at home and how the public perceives this issue should be investigated. Moreover, we aimed, through a web-based approach, to investigate and assess the community awareness of and commitment to stay-at-home orders during COVID-19 pandemic in Damietta Governorate, Egypt. Our findings provide insights into and data support for tailored interventions regarding community concerns and readiness to positively participate in preparedness efforts for the COVID-19 pandemic and to increase voluntary public compliance with social distancing and stay-at-home orders.

## Methods

This survey was part of a wider project targeting public health aspects relevant to the COVID-19 pandemic.

### Study design

A web-based cross-sectional survey using a snowballing sample was designed to assess community awareness of and commitment to stay-at-home orders during the COVID-19 pandemic in Damietta Governorate, Egypt.

### Sampling

The minimum sample size required was 385 (set as 500) according to the Raosoft sample size calculator (http://www.raosoft.com/samplesize.html), based on a standard deviation set at 1.96 for a 95% confidence interval, a 5% margin of error, an anticipated response of 50%, and a total population size of 1,565,252 (according to a 2020 estimate, Central Agency for Public Mobilization and Statistics: https://www.capmas.gov.eg/Pages/populationClock.aspx#).

### Population and inclusion criteria

The study sample comprised 500 participants from the general population in Damietta Governorate aged ≥ 18 years, considering gender weighing and geographical districts as possible, not diagnosed with or suspected to have COVID-19 symptoms (no close contacts and no fever or cold symptoms within the previous 2 weeks), who were social media users and agreed to participate in this survey. A stay-at-home measure was considered to have been applied when the population was advised by health authorities to voluntarily isolate at home for at least 2 weeks to prevent this infectious disease [10].

### Study tool and scoring system

We developed a web-based questionnaire to achieve wide, rapid distribution and avoid social contact. The duration of this study was from April 10, 2020, until the desired sample size that fulfilled the inclusion criteria was reached on July 15, 2020. The questionnaire was prepared in Arabic according to the WHO guidelines and the Egyptian MOH guidelines [11, 12]. The initial draft was sent to a group of multidisciplinary specialists in related fields to authenticate and validate the questions in terms of relativity, simplicity, and significance. Next, a reliability analysis was conducted to determine the internal consistency of the items. The Cronbach’s alpha reliability coefficient of the knowledge scale was 0.7. A pilot trial was conducted using 25 participants of various age groups and sex to test the comprehensibility of the questionnaire, estimate the time necessary to answer the questionnaire, and detect any necessary modifications. The pilot data were removed from the final analysis.

The questionnaire queried the participants to elicit the following information:

- Sociodemographic data; current or prior work in health fields, history of chronic illness, or seasonal flu vaccination; and whether they had experienced fever or cold symptoms within the last 2 weeks (removed from current analysis).
- Sources of knowledge on COVID-19: reliable sources (health care workers “HCWs,” the MOH, and the WHO) were considered when participants chose at least one of them.
- Participants’ knowledge of methods of COVID-19 prevention was assessed using a 15-item three-way (*yes*, *no*, *I do not know*) scale. The questionnaire inquired about the frequency of hand washing; avoiding crowds; hand greetings and touching the face with the hands; wearing a facemask in crowds; personal hygiene; using a tissue while coughing or sneezing; using the elbow to cover the face if tissue was not available; safe disposal of used masks, gloves, and tissues; and possible infection by asymptomatic patients. Correct answers received 1 point, and incorrect/I do not know answers received no points. The total score was 15. Participants with scores ≥ 12 points, from 8–11 points, and < 8 points were considered to have good, fair, or poor knowledge, respectively. The knowledge score was categorized based on modified Bloom’s cutoff points: poor (< 50%), fair (50–< 80%), and good (≥ 80%).
- Public response and commitment to stay-at-home orders during the previous 2 weeks. We categorized participants into two groups: stay-at-home responders (individuals who did not leave home at all or left for 1–2 days per week) and stay-at-home nonresponders (individuals who left home 3 days or more per week). We asked the participants how many times they left home per week, their average hours outside the home per day, who should stay-at-home, and their main reasons for leaving home.
- Public trust in governmental measures, community resources, and emergency services to manage the pandemic was tested. The following statements were used: “My local government & leaders work well (transparency, credible information, social and financial support, public engagement, quality of care provided, etc),” “My community has sufficient resources (capital, technology, materials, services, etc),” and “My community can provide emergency services (mutual aid groups, community emergency medicine, community solidarity, addressing the needs of disabled and elderly individuals, etc).” Their responses were measured by a 5-point Likert scale and graded as *strongly agree and agree* (good trust), *uncertain* (doubted trust), and *disagree and strongly disagree* (poor trust).

The web-based questionnaire was sent by the deanship office to various governmental and private sectors within Damietta. In addition, personal communications helped rapidly distribute the survey and broadcast it on the Internet through national websites and social media platforms. All participants could see the questionnaire, and they answered the questions by clicking the relevant link.

To overcome the possibility of weak or improper responses from participants, we used several methods verified in the literature, such as providing a cover letter, clear instructions, and follow-up reminders; asking participants to distribute the questionnaire link to family, relatives, and friends; and using a pre-notification regarding the aim of the survey, a plain design, and easy-to-read formats.

### Ethical approval

We obtained ethical approval from Damietta Faculty of Medicine IRB, Al-Azhar University (IRB no. 00012367-20-3-007; 21/3/2020). An anonymous electronic informed consent was added as an initial page before the online survey started, with emphasis on voluntary participation and withdrawal at any time without providing any justification.

### Statistical analysis

We conducted the statistical analysis using SPSS version 25.0 (IBM SPSS, Armonk, NY: IBM Corp., USA). For descriptive statistics, the mean (± SD) was used for quantitative variables, and the number and percentage were used for qualitative variables. For analytic statistics, the chi-square test or Fisher’s exact test was used to assess the differences in frequency of qualitative variables, and the Mann–Whitney test was applied for nonparametric data to assess the differences in means of quantitative variables. The statistical methods were verified, assuming a significance level of *p* < 0.05.

## Results

The mean age of study participants was 42.6 (± 14.8), from 18 to 72 years, 59% were females; 73.8% were married, 70.6% had university or higher education, and approximately three-fourths (74.8%) were working. The vast majority (98.8%) did not receive a seasonal flu vaccine, and 65.8% were not affected by chronic diseases. Approximately, 18.4% was stay-at-home responders (2% did not leave home and 16.4% left 1–2 days per week), and approximately, 81.6% were stay-at-home nonresponders (4% left home 3–4 days per week, and 77.6% left 5–7 days per week). Participants’ response to stay-at-home orders was stratified by each sociodemographic variable. The responses were significantly higher among elderly participants and those with a history of chronic illness; nonresponse was significant among participants who were married and working and those with a low family income (< 3000 pounds per month) (Table 1).

**Table 1:** General characteristics of studied population in Damietta Governorate, Egypt, during COVID-19 pandemic (April 10 to July 15, 2020)

Variables	Total ***n*** = 500 (%)	Stay-at-home responders ***n*** = 92 (%)	Stay-at-home nonresponders ***n*** = 408 (%)	***p***-value
Age (years) mean (± SD)	42.6 (± 14.8)	48.2 (± 15.8)	41.4 (± 14.3)	< 0.001*
Gender (female)	295 (59.0)	46 (50.0)	249 (61.0)	0.060
Marital status (married)	369 (73.8)	56 (60.9)	313 (76.7)	0.002*
Education (university or higher)	353 (70.6)	67 (72.8)	286 (70.1)	0.704
Working status (working)	374 (74.8)	23 (25.0)	351 (86.0)	< 0.001*
Residence (urban)	226 (45.2)	47 (51.1)	179 (43.9)	0.246
Family income/month (< 3000 pounds)	354 (70.8)	50 (54.3)	304 (74.5)	< 0.001*
Smoking habits (nonsmokers)	424 (84.8)	82 (89.1)	342 (83.8)	0.260
Work in health field^a^ (yes)	71 (14.2)	8 (8.7)	63 (15.4)	0.100
Seasonal flu vaccine (no)	494 (98.8)	90 (97.8)	404 (99.0)	0.305
History of chronic illness (yes)	171 (34.2)	63 (68.5)	108 (26.5)	< 0.001*

^a^Includes current or previous work. Values present as number and % were analyzed by Fisher’s exact test or chi-square test. Values present as mean ± SD were analyzed by Mann–Whitney *U*-test.*Significant

**Fig. 1:**
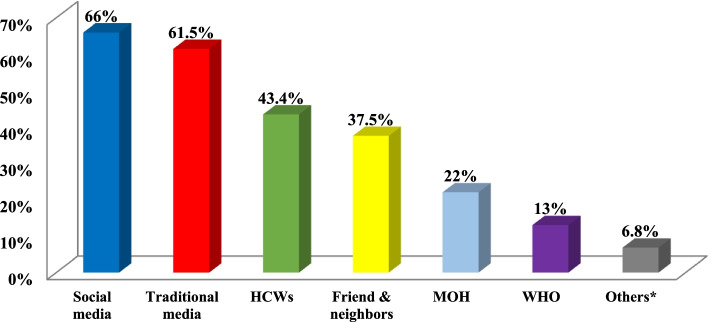
Sources of information on COVID-19 among studied population in Damietta Governorate, Egypt, during COVID-19 pandemic (April 10 to July 15, 2020). *Others include spouse, family members, coworkers, community leaders, newspaper, Google Search)

The sources of information on COVID-19 are illustrated in Fig. 1. Reliable sources, such as HCWs, the MOH, and the WHO, were represented by 43.4%, 22%, and 1%, respectively, and approximately two-thirds (66%) depended on social media.

**Fig. 2:**
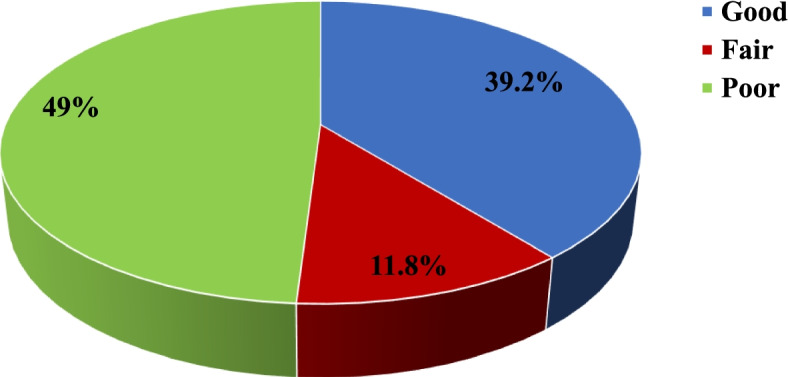
Overall knowledge score among studied population in Damietta Governorate, Egypt, during COVID-19 pandemic (April 10 to July 15, 2020)

The overall mean knowledge score was 8.66 (± 4.29). More than one-third (39.2%) of the participants had good knowledge of the effective methods of COVID-19 prevention, and 11.8% and 49% had fair and poor knowledge, respectively (Fig. 2).

Of the participants, more than 75% had good knowledge of frequent handwashing, avoiding crowds, wearing a facemask in crowds, personal hygiene, and possible infection by asymptomatic patients as effective methods of COVID-19 prevention. Stay-at-home responders had a significantly higher overall accepted knowledge (*p* < 0.001). The former were significantly more knowledgeable than the latter regarding the proper method of hand washing; avoiding touching the face with the hands; avoiding hand greetings; the safe disposal of used masks, gloves, and tissues; wearing a face mask in crowds; proper nutrition; personal hygiene; and possible infection by asymptomatic patients (Table 2).

**Table 2:** Correct responses of studied population in Damietta Governorate, Egypt, on knowledge items regarding effective methods of COVID-19 prevention during COVID-19 pandemic (April 10 to July 15, 2020)

Variables	Total ***n*** = 500 (%)	Stay-at-home responders ***n*** = 92 (%)	Stay-at-home nonresponders ***n*** = 408 (%)	***p***-value
Frequent hand washing	426 (85.2)	84 (91.3)	342 (83.8)	0.074
Proper method of hand washing	330 (66.0)	81 (88.0)	249 (61.0)	< 0.001*
Avoiding touching face with hands	251 (50.2)	83 (90.2)	168 (41.2)	< 0.001*
Avoiding hand greetings	138 (27.6)	67 (72.8)	71 (17.4)	< 0.001*
Using tissue while coughing or sneezing	248 (49.6)	54 (58.7)	194 (47.5)	0.065
Using elbow to cover face if tissue is not available	244 (48.8)	52 (56.5)	192 (47.1)	0.107
Safe disposal of used mask, gloves, and tissues	199 (39.8)	49 (53.3)	150 (36.8)	0.005*
Using mouth gurgleǂ	212 (42.4)	44 (47.8)	168 (41.2)	0.246
Taking antibioticsǂ	183 (36.6)	36 (39.1)	147 (36.0)	0.632
Avoiding crowds	432 (86.4)	83 (90.2)	349 (85.5)	0.312
Wearing a face mask in crowds	399 (79.8)	81 (88.0)	318 (77.9)	0.031*
Proper nutrition	348 (69.6)	74 (80.4)	274 (67.2)	0.012*
Personal hygiene	388 (77.6)	82 (89.1)	306 (75.0)	0.003*
Possible infection by asymptomatic patients	414 (82.8)	86 (93.5)	328 (80.4)	0.002*
Virus can stay on surfaces	119 (23.8)	28 (30.4)	91 (22.3)	0.105
Overall accepted knowledge	196 (39.2)	49 (53.3)	147 (36.0)	< 0.001*

Values presented as number and percent were analyzed by Fisher’s exact test or chi-square test. ǂReversed statements (not effective method of COVID-19 prevention). *Significant

The majority of participants preferred for elderly and symptomatic individuals to stay-at-home, 88.9% and 91.6%, respectively; approximately, 79.2% stayed outside the home for more than 5 h per day, and the main causes of leaving home during the last 2 weeks were buying essentials and food (86.2%) and going to work (74.8%), and 37% reported leaving home to meet friends and relatives (Table 3).

**Table 3:** Commitment to stay-at-home orders among studied population in Damietta Governorate, Egypt, during COVID-19 pandemic (April 10 to July 15, 2020)

Variables	Frequency ***n*** = 500	Percentage (%)
Who should stay-at-home^a^		
Elderly	446	88.9
Symptomatic	458	91.6
Pregnant	288	57.6
Children	199	39.8
All community members	4	0.8
Do not know	38	7.6
Average hours stayed outside home/day		
< 1 h	28	5.6
1–3 h	36	7.2
3–5 h	30	6.0
> 5 h	396	79.2
NA	10	2.0
Main cause of leaving home^a^		
Buy essentials, e.g., food	431	86.2
Go to work	374	74.8
Visit hospital or physician	27	5.4
Meet friends and relatives	185	37.0
Entertainment	36	7.2
No cases recorded nearby	43	8.6
NA	10	2.0

*NA*, Not applicable; did not leave home during the last 2 weeks. ^a^Questions with more than one answer

The majority of participants had poor trust in governmental measures, community resources, and emergency services to manage the pandemic, 84.6%, 71.8%, and 79%, respectively, with no significant differences between responders and nonresponders to stay-at-home orders: *p* = 0.113, *p* = 0.513, and *p* = 0.273, respectively (Table 4).

**Table 4:** Trust in governmental measures, community resources, and emergency services among studied population in Damietta Governorate, Egypt, during the COVID-19 pandemic (April 10 to July 15, 2020)

Variables		Total ***n*** = 500 (%)	Stay-at-home responders ***n*** = 92 (%)	Stay-at-home nonresponders ***n*** = 408 (%)	***p***-value
My local government and leaders work well	Good	13 (2.6)	5 (5.4)	8 (2.0)	0.113
	Doubted	64 (12.8)	14 (15.2)	50 (12.3)	
	Poor	423 (84.6)	73 (79.3)	350 (85.8)	
My community has sufficient resources	Good	33 (6.6)	8 (8.7)	25 (6.1)	0.513
	Doubted	108 (21.6)	22 (23.9)	86 (21.1)	
	Poor	359 (71.8)	62 (67.4)	297 (72.8)	
My community can provide emergency services	Good	34 (6.8)	8 (8.7)	26 (6.4)	0.273
	Doubted	71 (14.2)	17 (18.5)	54 (13.2)	
	Poor	395 (79.0)	67 (72.8)	328 (80.4)	

Values presented as number and percent were analyzed by chi-square test

## Discussion

Because of the difficulties in timely vaccine production and distribution, control measures to reduce public contact, such as social “physical” distance, self-isolation (SI), self-quarantine, home isolation, and staying at home, were considered, in addition to hygienic measures, to hamper the pandemic influenza transmission. The effectiveness of this strategy largely depends on public compliance with public health containment and intervention measures, and compliance was shown to increase significantly as the understanding of pandemic influenza increased [8].

### Sociodemographics and responses to stay-at-home orders

Analysis of the characteristics of individuals regarding their response to preventive measures is important and to directing social assistance to those most in need. Personal behavior during an epidemic depends, in part, on sociodemographics [13]. Our results found an effect of sociodemographic variables on participants’ response to stay-at-home orders, which was significantly higher among elderly individuals and those with a history of chronic illness, and nonresponders were more likely to be married, working, and have low family income. Zhang et al. identified age, gender, marriage, and education as significant variables that affect Chinese willingness to practice SI when experiencing a pandemic risk [13].

The significant difference in working status between responders and nonresponders implies that employment-related constraints may have a major impact on implementing social distancing.

Many researchers have discussed the influence of socioeconomic factors on control measures during epidemics, including gender differences [14], higher age [15], education [16], perception of the epidemic in the community [13], marital status [17], and living with individuals in need of special care who have a high risk of infection [18]. Our findings did not demonstrate gender or education differences between responders and nonresponders. Contrary to our results, women may suffer higher stress and depression levels than men during pandemics, and those phenomena may affect women’s response to control measures. This difference may be related to their different exposures to health risks and greater vulnerability to health risks than men, in addition to women’s disproportionate burden to perform domestic responsibilities [19]. The lower the education level of a group, the less information they may receive about the pandemic, and the lower their likeliness to understand the importance of these measures [16]. We also found that nonresponders were more likely to be married than responders; however, married individuals may enjoy better physical and mental health than non-married individuals, which affects the former’s behavior and favors their commitment to control measures [17].

### Commitment to stay-at-home orders

The participants (98%) left home for different reasons, mainly buying essentials and food (86.2%) and going to work (74.8%). Similarly, in the UK, shopping for groceries, including food, was one of the main reasons individuals reported for breaking SI [20]. By contrast, in Saudi Arabia, 46.9% of the population left home to buy necessities, and 12.8% left to go to work or hospital [21]. Health beliefs conceptualized by the health belief model, including susceptibility to COVID-19, perceived severity of the disease, perceived barriers to staying at home, and having the intention to perform the recommended health behavior (stay-at-home), are possible explanations for compliance with stay-at-home orders when experiencing pandemic risk [22].

Understanding how to devise, present, and implement social distancing measures, including staying at home, such that they are acceptable to the public, is important in planning and responding to infectious disease epidemics. In applying the widespread restrictions during the COVID-19 pandemic, challenges have been identified, including the population’s compliance with these measures, how to monitor the health status of individuals who stayed at home or self-isolated/quarantined, how to provide their essential needs, secondary health effects on vulnerable groups, and the consequences of economic losses, social discrimination, boredom and monotony, and uncertainty about the future [23–25].

Many participants (81.6%) opposed stay-at-home orders, which intensifies the need to understand how social distancing is perceived in Egypt. In contexts of infectious disease epidemics, strong public support for the control measures has been proven in high-income countries [26–28]; however, our results and those in low-income countries show attitudes of noncompliance [29, 30].

Various issues may affect compliance with and engagement in control measures during epidemics, including interpersonal, environmental, academic, and social factors and risk perception [31–33]. The diversity of individuals’ concerns and needs should be considered in interventions aiming to increase voluntary compliance with control measures and widespread restrictions. Kpanake et al. discussed the acceptability of community quarantine during the Ebola epidemic in West Africa. They found that for 18% of the participants, quarantine was never acceptable; for 14%, it depended on the level of contagiousness and lethality; and for 36%, their judgment depended on the availability of support services for quarantined individuals [29]. Hong et al. discussed differences in stay-at-home behaviors in China and the USA during peaks of the COVID-19 pandemic: participants in the USA perceived high levels of susceptibility, participants in China perceived high levels of severity, and both perceptions increased the frequency of stay-at-home behaviors [22].

### Trust in governmental measures, community resources, and emergency services

Community preparedness and resources, public engagement, and trust in authorities, governmental action, and emergency services are among the other variables affecting response to stay-at-home orders and are vital for successful interventions during a pandemic [34, 35]. The majority of our participants (84.6%) experienced poor trust in governmental measures. This distrust might be explained by their concern about vaccine availability and effectiveness, appropriateness of care provided by health authorities, and governmental support during the early periods of the pandemic. Distrust may impede health initiatives and programs for infectious disease prevention. In the same context, where distrust prevails, trust-building actions, such as defining rights and obligations, prioritizing “the greater good,” and increasing transparency, are prone to failure. Thus, authorities should exhibit managerial preparedness and competence, engage in intense efforts to inform the public of the seriousness of the pandemic threat, market the need for compulsory policies, and seek approval for these policies from independent scientists, professional groups, and opinion leaders [36, 37].

Zhang et al. suggested that trusted leadership and government and the availability of effective emergency services have significant positive impacts on accepting control measures and promotes the willingness to SI [13].

To rebuild the public’s trust, local governments should improve their communication with their constituents, promote relationships, work with formal and informal leaders, and call for commitment from community organizations and leaders. Several factors should be considered to encourage the public to accept widespread restrictions and control measures related to a pandemic and to increase compliance with public health containment measures. First, to increase public awareness regarding pandemic risks, authorities should conduct extensive publicity in various forms (e.g., trusted spokespeople) to educate the public on the pandemic, stress the benefits of compliance, and reiterate the importance of everyday protective measures to raise their awareness when facing a pandemic risk [13, 38]. However, some studies have questioned the role of publicity in solving the problem or reducing the number of infections [39]. Therefore, an important measure is for the government to establish an early warning system to provide correct information on pandemics and to maintain communication with the public. Second, when there is a pandemic risk, to improve public health social norms, the government, civil society, and the media should cooperate in communicating to the public, and the government should formulate relevant regulations to regulate behavior. Third, public supervision, participation, and empowerment should be encouraged. Fourth, endeavors to increase perceived self-efficacy and provide sufficient public training to decrease the difficulty of applying health behaviors should be attempted. Fifth, the government should build trust with its constituents so that the public will make the sacrifices necessary to implement the measures to diminish the transmission of the diseases [40, 41].

### Study limitations

This study has some limitations: (1) Making causal inferences was difficult because a cross-sectional design was used. (2) The snowball method is a nonrandom sampling method; thus, the sample in this study is not representative of a district-level population and is not a random sample of the general population. (3) We used a web-based survey, which can lead to selection bias, an underestimation of the current situation, and limited participation of individuals, such as those who are elderly and poor and who are less likely to use technology (e.g., social media) than their younger and wealthier counterparts. (4) In the sample, health-oriented individuals and those highly concerned about the outbreak are possibly overrepresented. (5) The self-reported information may not be entirely accurate (due to recall bias); thus, it should be viewed with caution (social desirability bias). (6) We did not gain insights from investigating public perception, perceived barriers, and possible incentives regarding staying at home. (7) If the pandemic continues, compliance with protective measures may diminish; thus, caution should be used when generalizing the results. (8) Residual confounding caused by unmeasured covariates possibly occurred, and the findings may vary in other populations with different cultural, ethnic, and geographical backgrounds.

## Conclusions

Participants’ compliance with and engagement in stay-at-home orders in Damietta Governorate, Egypt, was poor. Public response to stay-at-home orders is affected by sociodemographics, and the public’s trust in governmental measures, community resources, and emergency services was poor. Understanding how social distancing is perceived in Egypt is important to provide public support and improve pandemic disease containment.

## Data Availability

The datasets used and analyzed in this study are available from the corresponding author on reasonable request. Confidentiality and security of data and materials were ensured through all stages of the study.

## Abbreviations

SARS: Severe acute respiratory syndrome
WHO: World Health Organization
MOH: Ministry of Health
CDC: Centers of Disease Control and Prevention
SI: Self-isolation
HCWs: Healthcare workers
